# Physical Exercise as an Adjuvant to Glucocorticoid Therapy: Clinical Recommendations and Evidence Review

**DOI:** 10.1007/s40279-026-02469-6

**Published:** 2026-06-08

**Authors:** Paula Etayo-Urtasun, Mikel L. Sáez de Asteasu, Mikel Izquierdo

**Affiliations:** 1 Navarrabiomed, Hospital Universitario de Navarra (HUN)-Universidad Pública de Navarra (UPNA), IdiSNA, Pamplona, Spain; 2 CIBER of Frailty and Healthy Aging (CIBERFES), Instituto de Salud Carlos III, Madrid, Spain; 3 Department of Health Sciences, Public University of Navarre, 31008 Pamplona, Spain

## Abstract

Glucocorticoids are extensively prescribed for managing immune-mediated and inflammatory diseases. While clinically effective, prolonged glucocorticoid therapy induces adverse effects that compromise physical function and may attenuate the benefits of exercise interventions. Current exercise guidelines, however, inadequately address the complexities of concurrent glucocorticoid treatment. This narrative review, which includes a systematic review, examines interactions between glucocorticoid therapy and physical exercise, evaluates existing evidence on exercise interventions in glucocorticoid-treated populations, and provides evidence-based exercise recommendations tailored to this clinical population. Glucocorticoid-induced complications—including diabetes mellitus, musculoskeletal disorders, hypertension, weight gain, cognitive impairment, and neuropsychiatric disturbances—substantially impair exercise capacity, patient engagement, and physiological adaptations to training. After searching PubMed, Scopus, and Web of Science databases from 1 January, 2000 to 10 May, 2025 for exercise interventions in glucocorticoid-treated patients, 19 studies were included (18 unique trials; *n* = 663, predominantly women), mainly involving rheumatoid arthritis and systemic lupus erythematosus. Overall, the methodological quality, as assessed using the Risk of Bias 2 tool, was rated as moderate to high. Exercise interventions consistently improved muscle performance and functional outcomes, whereas evidence regarding bone, cardiometabolic, cognitive, and mental health outcomes was limited or inconsistent. For optimal exercise prescription in this population, resistance training and aerobic exercise emerge as essential components, complemented by targeted strategies to enhance adherence and ensure safety. Integrating structured exercise as adjuvant therapy in long-term glucocorticoid treatment holds considerable promise for mitigating adverse effects and reducing polypharmacy burden; however, additional research is warranted to establish definitive clinical guidelines.

**Supplementary Information:**

The online version contains supplementary material available at https://doi.org/10.1007/s40279-026-02469-6.

## Key Points

**Table:** 

Glucocorticoid therapy induces multiple adverse effects—including diabetes mellitus, musculoskeletal disorders, hypertension, weight gain, cognitive impairment, and neuropsychiatric complications—that collectively compromise exercise capacity, patient engagement, and physiological adaptations to physical training.
Exercise interventions demonstrate significant benefits for muscle performance and physical function in patients receiving glucocorticoid therapy; however, evidence regarding their effects on bone health, body composition, cardiovascular outcomes, metabolic parameters, mental health, and cognitive function remains limited and requires further investigation.
Current evidence supports implementing individualized exercise prescriptions that incorporate progressive resistance training and aerobic conditioning, supplemented by targeted strategies to optimize patient adherence and ensure safety throughout the intervention period.
Integrating structured exercise programs as adjunctive therapy in patients requiring long-term glucocorticoid treatment represents a critical strategy for mitigating adverse effects, reducing polypharmacy burden, and advancing a comprehensive, patient-centered approach to chronic disease management.

## Introduction

In the era of precision medicine, integrating exercise with pharmacotherapy has evolved from an optional consideration to an essential component of comprehensive patient care. As the global burden of chronic diseases continues to expand, there is an increasing need for individualized interventions that extend beyond traditional pharmacological prescriptions. When appropriately prescribed, exercise not only complements drug therapy but also enhances medication efficacy, reduces adverse drug reactions, and mitigates the risks associated with polypharmacy [1]. Despite this therapeutic potential, current exercise guidelines rarely account for patients' pharmacological profiles, particularly those receiving long-term medication regimens [2].

This oversight is particularly pronounced in the management of patients receiving synthetic glucocorticoids—cornerstone therapeutic agents used across a broad spectrum of immune and inflammatory disorders, including asthma, chronic obstructive pulmonary disease (COPD), inflammatory bowel disease, rheumatic conditions, and various malignancies [3]. Glucocorticoids are prescribed to approximately 1–3% of adults each year in high-income countries, with oral formulations used by more than 1% of adults; use rises with age, and a substantial proportion remain on treatment beyond 2 years [4, 5].

While highly effective in suppressing inflammatory responses, glucocorticoids are associated with a diverse array of adverse effects that critically intersect with the physiological systems governing physical performance and exercise adaptation [6, 7]. These complications include diabetes mellitus, musculoskeletal disorders (osteoporosis, osteonecrosis, myopathy, and sarcopenia), hypertension, weight gain, cognitive impairment, and neuropsychiatric symptoms [3]. Such complications may substantially reduce physical capacity, interfere with normal physiological responses to exercise, and/or diminish adherence to structured exercise programs.

Although glucocorticoids disrupt tissue homeostasis through multiple pathways, several inter-related nodes unify these complications: (1) systemic insulin resistance with ectopic/visceral adiposity that worsens glycemia, dyslipidemia, and blood-pressure control; (2) skeletal-muscle catabolism and mitochondrial dysfunction (suppressed protein synthesis, enhanced proteolysis, type II fiber atrophy, impaired oxidative phosphorylation) driving myopathy and exercise intolerance; (3) endothelial dysfunction and autonomic imbalance contributing to hypertension and reduced exercise tolerance; (4) suppressed osteoblastogenesis and altered bone remodeling predisposing to osteoporosis/fragility; and (5) neurocognitive vulnerability (hippocampal/prefrontal effects) underpinning mood, motivation, and executive dysfunction (Table 1) [6, 8–11]. These nodes are precisely the targets of exercise training: combined aerobic and resistance training improves insulin sensitivity and reduces cardiometabolic risk; progressive resistance and power training counteract muscle catabolism and restore mitochondrial function; aerobic exercise enhances endothelial function and autonomic balance; mechanical loading stimulates osteogenesis; and multicomponent or cognitively enriched programs support neurotrophic signaling and cognition [2].

**Table 1:** Unifying mechanisms and exercise countermeasures in long-term glucocorticoid therapy

Convergent mechanism	Dominant clinical expressions	Exercise countermeasure (dose/theme)
Insulin resistance and ectopic fat	Hyperglycemia, dyslipidemia, hypertension	Combined aerobic (≥ 150 min/wk moderate-intensity) + resistance training (2–3 sessions/wk); consider post-prandial sessions; break up sedentary time
Muscle catabolism and mitochondrial dysfunction	Myopathy, sarcopenia, fatigue, reduced power	Progressive resistance training (whole-body, 2–3 × /wk, 40–60%1RM progressing); power sets for II fibers; ensure protein sufficiency
Endothelial dysfunction and autonomic imbalance	Exercise intolerance, elevated BP	Moderate-intensity aerobic most days; extended cool-down; avoid Valsalva during resistance training; monitor blood pressure
Suppressed osteoblastogenesis and altered remodeling	Low BMD, fractures, vertebral deformity	Resistance + balance training; carefully dosed impact loading when safe; spine-sparing technique
Cortical/hippocampal vulnerability and mood/anxiety	Cognitive complaints, low motivation, anxiety/depression	Multicomponent training (aerobic + resistance + dual-task/power); consider group-based/supervised formats; adjunct exergaming

*1RM* one-repetition maximum, *BMD* bone mineral density, *BP* blood pressure, *wk* week

Nevertheless, most contemporary exercise prescription guidelines remain largely agnostic regarding concurrent glucocorticoid therapy. This lack of integration between pharmacological treatment considerations and exercise recommendations significantly limits our capacity to deliver safe, effective, and truly individualized care. As evidence continues to accumulate supporting the therapeutic role of physical activity in chronic disease populations, it becomes increasingly critical to understand how glucocorticoid therapy modulates both the potential risks and the benefits of exercise interventions.

This narrative review, which includes a systematic review section, aims to address this important knowledge gap. We first explore how glucocorticoids influence exercise capacity, patient engagement, and physiological adaptation to physical training. Then, we synthesize findings from clinical trials that have studied the effects of exercise interventions in glucocorticoid-treated populations, especially focusing on patients with immune-mediated inflammatory diseases. The synthesis of the included clinical trials was performed using a systematic review with a qualitative synthesis, following the PRISMA 2020 guidelines. Given heterogeneity in populations, interventions, and outcomes, meta-analysis was not planned. Finally, we offer a comprehensive set of evidence-based recommendations to guide exercise prescription in this clinical setting, thereby supporting the safe, effective, and personalized integration of exercise therapy into existing pharmacological treatments.

## Influence of Glucocorticoid-Induced Adverse Effects on Exercise Capacity, Engagement and Adaptations

Exercise capacity reflects the combined ability of the cardiovascular, respiratory, neuromuscular, and metabolic systems to perform physical work, often measured by peak oxygen uptake (VO_2_peak), submaximal tests such as the 6-min walk test (6MWT), Timed Up and Go (TUG), gait speed, and sit-to-stand tests, and muscle strength or power (e.g., handgrip). Cross-sectional studies indicate that glucocorticoid-treated groups have lower strength and shorter 6-min walk distance (6MWD) compared to age- and disease-matched counterparts [12, 13]. Physical activity refers to behaviors performed daily, such as minutes of moderate-to-vigorous physical activity (MVPA), step count, and sedentary time. Observational studies indicate that patients prescribed glucocorticoids have lower physical activity levels compared to non-users [14]. Together, these data show that glucocorticoids are linked to both behavioral restrictions (less MVPA/more sitting) and physiological deconditioning (lower strength and cardiorespiratory capacity), each of which requires specific exercise countermeasures. This emphasizes the importance of awareness of these factors for accurate initial assessment before starting any exercise intervention.

Although glucocorticoids are highly effective in treating various diseases, their long-term use can lead to significant side effects that severely affect physical function and participation in exercise. These side effects include insulin resistance and diabetes mellitus, musculoskeletal issues, hypertension, weight gain, cognitive problems, and neuropsychiatric conditions. Many of these symptoms arise from shared mechanisms such as insulin resistance, muscle breakdown/mitochondrial dysfunction, and endothelial dysregulation, which directly limit training ability and adaptation. These adverse effects can impair an individual’s exercise capacity while also decreasing adherence to exercise routines and reducing exercise-related physiological improvements.

### Glucocorticoid Therapy and Exercise Capacity

Multiple adverse effects of glucocorticoid therapy can significantly impair exercise capacity, making awareness of these factors crucial for accurate initial assessment before implementing any exercise intervention. Glucocorticoid-induced osteoporosis, recognized as the leading cause of secondary osteoporosis, develops through disruption of the normal balance between bone formation and resorption [6, 15]. Glucocorticoids suppress osteoblastogenesis (down-regulating the Wingless-related integration site (Wnt)/β-catenin and runt-related transcription factor 2 (RUNX2)), increase osteoblast/osteocyte apoptosis, and shift the receptor activator of nuclear factor κB ligand (RANKL)/osteoprotegerin (OPG) axis toward osteoclastogenesis; they also reduce intestinal calcium absorption, increase renal calcium loss, and suppress gonadal steroids, collectively resulting in low bone formation and high bone resorption [15]. Clinically, long-term use is associated with lower bone mineral density (BMD) and vertebral microarchitectural deterioration, especially in chronic inflammatory diseases [16]. These skeletal manifestations carry substantial clinical significance, as reduced BMD translates to markedly elevated fracture risk, with 30–50% of long-term glucocorticoid users sustaining at least one fracture during treatment [17]. Consequences relevant to exercise include pain, kyphosis, impaired balance and mobility, and fear of falling, which narrow the safe exercise envelope and may blunt responsiveness to loading-based interventions [18].

Osteonecrosis represents another serious skeletal complication frequently observed in long-term glucocorticoid users, especially those receiving high cumulative doses [19]. Prevalence estimates range from 9 to 40%, with hip involvement being the most commonly affected site [20]. Glucocorticoid-related osteonecrosis involves ischemic injury caused by marrow adipocyte hypertrophy, increased intraosseous pressure, fat embolism, hypercoagulability, microthrombosis, impaired angiogenesis (such as reduced vascular endothelial growth factor (VEGF) signalling), and osteocyte apoptosis. These changes disrupt subchondral blood flow and healing, increasing the risk of the femoral head collapsing during weight bearing. Patients with glucocorticoid-induced osteonecrosis typically experience significant joint pain that progressively limits their range of motion and functional capacity [21]. Furthermore, osteonecrosis substantially increases the risk of joint collapse during weight-bearing activities, creating additional constraints on exercise prescription and potentially compromising overall exercise capacity [22].

Glucocorticoid-induced hypertension, another well-documented adverse effect reported extensively in clinical studies [9], contributes significantly to the already elevated cardiovascular risk-profile characteristic of immune-mediated inflammatory conditions [23]. Hypertension is associated with reduced cardiovascular tolerance to physical exertion, manifesting as early-onset fatigue and decreased exercise performance [24]. Given the substantial hemodynamic burden imposed by elevated blood pressure, patients require close monitoring and carefully tailored exercise prescriptions to ensure safety and optimize therapeutic outcomes [25].

Cognitive dysfunction represents an additional potential consequence of glucocorticoid therapy that may substantially impair exercise capacity and participation [3, 26]. Glucocorticoid exposure has been associated with measurable declines in both declarative and working memory, cognitive domains that play critical roles in understanding and successfully following exercise instructions [11]. Moreover, glucocorticoid-induced cognitive dysfunction adversely affects attention span and executive function, thereby compromising decision-making capabilities and impairing learning processes essential for effective exercise program implementation [26].

### Glucocorticoid Therapy and Exercise Engagement

Specific adverse effects induced by glucocorticoid therapy can significantly impede patient participation in exercise programs [27]. Recognition of these barriers is essential for developing and implementing effective strategies to enhance adherence to exercise interventions among glucocorticoid-treated patients.

Neuropsychiatric complications represent a major category of exercise-limiting adverse effects. Multiple cohort studies have documented higher rates of anxiety symptoms in glucocorticoid users [26]. Patients experiencing glucocorticoid-induced anxiety frequently report fears associated with physiological arousal sensations, including tachycardia and dyspnea, which commonly lead to deliberate avoidance of physical exertion [28]. This avoidance behavior subsequently results in diminished tolerance to moderate- and high-intensity exercise [29]. Depression presents an equally significant barrier, with glucocorticoid treatment conferring a twofold increased risk of depressive episodes (prevalence of 11.1% in glucocorticoid users compared to 4.1% in non-users) [30, 31]. Individuals with glucocorticoid-induced depression characteristically exhibit reduced intrinsic motivation and persistent apathy, factors that contribute to negative perceptions of exercise and decreased participation in physical activity programs [32].

More severe neuropsychiatric manifestations associated with high-dose or prolonged glucocorticoid therapy pose additional challenges to exercise engagement. A large-scale epidemiological study of 372,696 patients demonstrated approximately sevenfold higher rates of suicide attempts or completion and four- to fivefold increased risks of developing mania or delirium among glucocorticoid users [30]. These profound psychological disturbances further compromise intrinsic motivation, treatment adherence, and the capacity to derive satisfaction from exercise participation. Consequently, behavioral interventions incorporating personalized goal setting and robust social support systems are critical components of successful exercise programs for this population [33].

Metabolic alterations, particularly glucocorticoid-induced weight gain and increased visceral adiposity, constitute another significant barrier to exercise adherence [34]. These changes result from fundamental alterations in lipid metabolism, characterized by enhanced lipolysis and adipogenesis that preferentially redistribute adipose tissue to central anatomical sites [35]. From a behavioral standpoint, body image dissatisfaction and diminished self-efficacy associated with these physical changes can substantially impair patient engagement in structured exercise programs [36]. Understanding and addressing these multifaceted barriers is therefore crucial for optimizing exercise interventions in glucocorticoid-treated patients.

### Glucocorticoid Therapy and Exercise Adaptations

Glucocorticoid-induced myopathy represents the most prevalent form of drug-induced muscle disorder [37]. These agents exert profound catabolic effects on skeletal muscle by suppressing protein synthesis while simultaneously promoting proteolysis, ultimately resulting in type II fiber atrophy and progressive functional decline [8]. Chronic glucocorticoid exposure induces oxidative stress and mitochondrial dysfunction within skeletal muscle tissue, leading to excessive lactate production and compromised muscle performance [38]. Furthermore, prolonged glucocorticoid administration significantly reduces muscle strength, thereby potentially attenuating exercise-induced physiological adaptations [8]. Even short-term glucocorticoid exposure has been demonstrated to impair sarcolemma excitability and reduce muscle fatigue thresholds [39]. The resultant decrease in muscle mass and strength substantially increases the risk of falls and fractures, highlighting the critical importance of implementing individualized, progressive resistance training (PRT) protocols [40].

Glucocorticoid-induced diabetes mellitus represents another clinically significant adverse effect with substantial implications for exercise adaptation, accounting for up to 2% of all type 2 diabetes cases encountered in primary-care settings [41]. These metabolic perturbations may compromise adaptive responses to exercise through several distinct mechanisms. In type 2 diabetes, intracellular pathways governing glucose uptake become impaired, resulting in slower glucose uptake and glycogen resynthesis [10]. A second critical mechanism involves impaired endothelial function, arising from the deleterious interaction between advanced glycation end products and reactive oxygen species, which leads to reduced endothelium-dependent vasodilation, restricted muscle blood flow during exercise, and ultimately blunted exercise adaptations [42]. Additionally, the chronic systemic inflammation and oxidative stress characteristic of type 2 diabetes interfere with exercise-induced anabolic signaling pathways, thereby inhibiting muscle regeneration and limiting the development of muscle mass and strength [43]. The combination of impaired glucose metabolism and altered body composition results in reduced aerobic efficiency and altered energy balance responses, which collectively hinder both training load progression and adaptations to physical exercise [10, 35].

Glucocorticoid-induced suppression of bone turnover further compromises the osteogenic response to mechanical loading [15]. In osteoporotic conditions, osteocyte sensitivity to mechanical stimuli becomes markedly reduced due to multiple cellular mechanisms, including signal desensitization, osteocyte apoptosis, and impaired mechanotransduction pathways [44]. These pathophysiological alterations result in substantially diminished bone adaptation in response to exercise interventions.

**Fig. 1:**
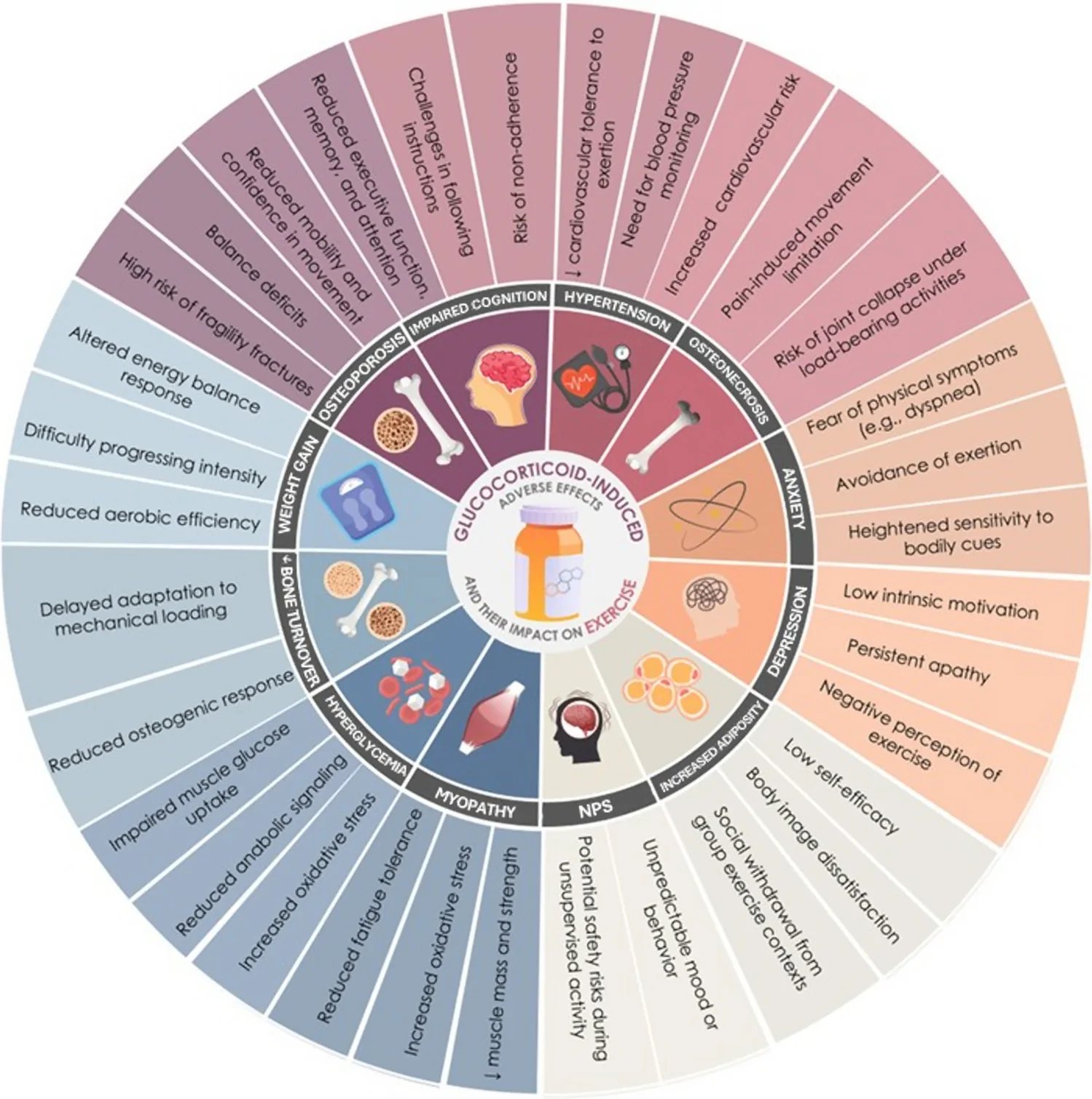
Glucocorticoid-induced adverse effects and their impact on exercise. *NPS* neuropsychiatric symptoms

Supplementary Table S1 (Electronic Supplementary Material, ESM) provides a comprehensive summary of glucocorticoid-induced adverse effects and their complex interactions with exercise capacity, engagement, and adaptation. Figure 1 presents a graphical representation of the multifaceted interactions between glucocorticoid treatment and exercise responses. The methodological approach for selecting evidence regarding glucocorticoid-induced adverse effects is detailed in ESM Methods S1.

## Evidence for Exercise Interventions in Glucocorticoid-Treated Populations

### Study Selection

**Fig. 2:**
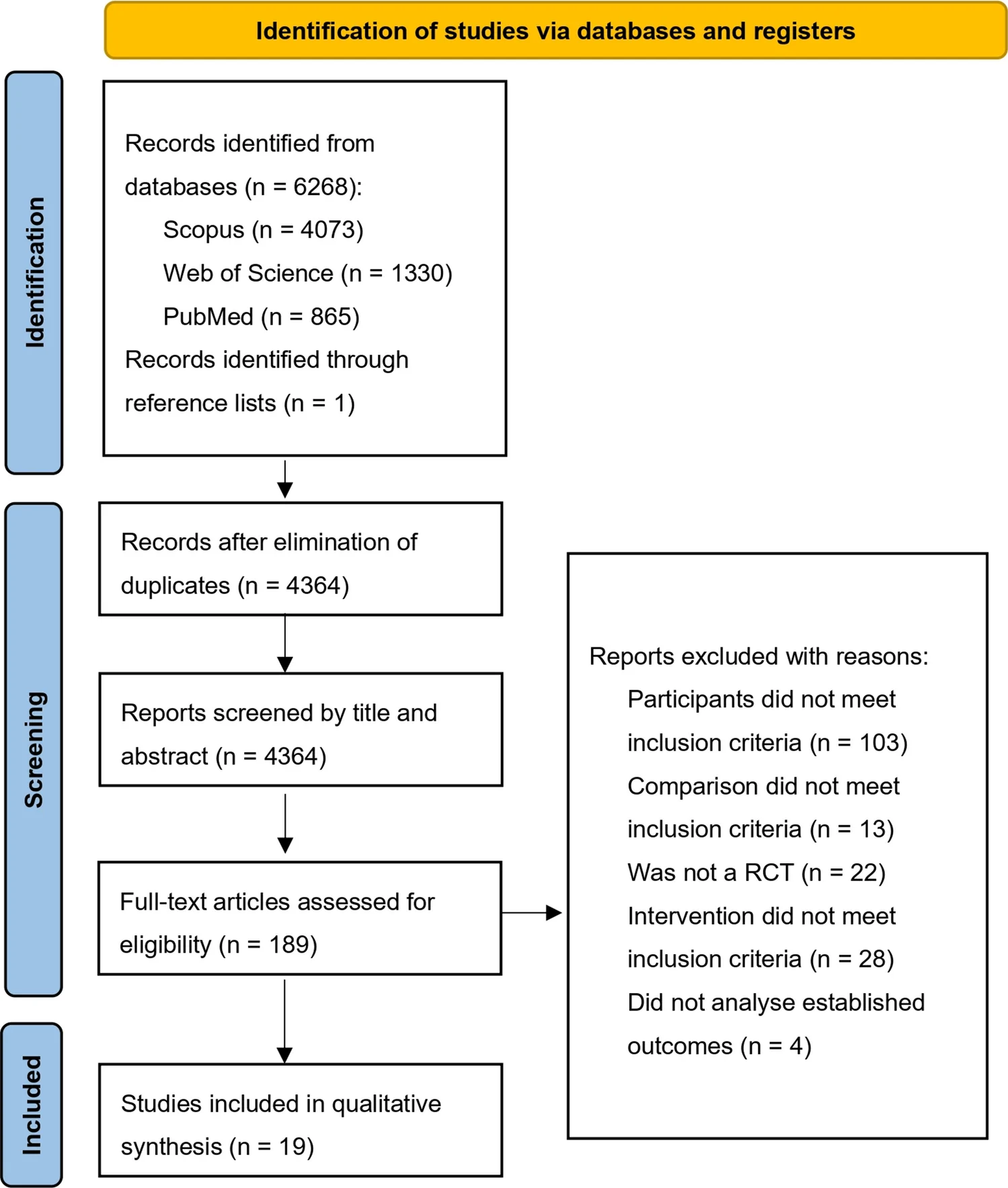
Study identification and selection flow diagram. *n* number of reports, *RCT* randomized controlled trial

The review protocol was registered in PROSPERO (CRD420251049238). The search strategy combined terms related to glucocorticoid exposure, exercise/physical training and study type (ESM Methods S2). Two reviewers independently screened titles, abstracts, and full texts, extracted data using a standardized template, and resolved any disagreements through consultation with a third reviewer. We included randomized controlled articles (RCTs) that assessed the effects of physical exercise interventions in adults with autoimmune diseases, at least half of whom were taking oral glucocorticoids. The study selection process is detailed in Fig. 2. Electronic database searches yielded 6,268 records: 4,073 from Scopus, 1,330 from Web of Science, and 865 from PubMed. Manual screening of reference lists identified one additional study, bringing the total to 6,269 records.

Following duplicate removal (*n* = 1,905), 4,364 unique titles and abstracts underwent initial screening. Based on predefined inclusion criteria, 189 full-text articles were selected for comprehensive evaluation. Application of the eligibility criteria resulted in the exclusion of 170 articles due to insufficient glucocorticoid-specific data, inadequate outcome reporting, or interventions that did not involve exercise. Ultimately, 19 studies [45–63] satisfied all inclusion criteria and were incorporated into the final qualitative synthesis (ESM Methods S2).

### Risk-of-Bias Assessment

The methodological quality evaluation of the included studies is presented in ESM Figs. S1 and S2. Overall, most studies raised some concerns regarding risk of bias, while several were rated as low risk and a few as high risk. Most RCTs provided clear descriptions of allocation procedures, blinding of outcome assessors, and missing-data management. Across trials, risk of bias was most frequently driven by unclear allocation concealment and incomplete reporting of adherence/progression.

### Participant Characteristics

The 19 included articles represented 18 distinct studies (one secondary analysis) encompassing 663 participants (595 women, 68 men). Nine studies enrolled exclusively female participants. The majority of trials focused on individuals with rheumatoid arthritis [48, 53, 57, 59, 60, 63], systemic lupus erythematosus [50, 52, 54], and polymyositis or dermatomyositis [45–47, 49, 61]. Additional studies investigated exercise interventions in patients with sarcoidosis [56, 58, 62], myasthenia gravis [55], and Sjögren’s syndrome [51]. Detailed participant characteristics and study demographics are presented in Table 2.

**Table 2:** Summary of qualitative analysis of the included studies

Study	Population	GC exposure %; median (IQR)	Supervision level	Intervention characteristics	Comparator	Measurement methods	Changes in IG compared to CG (Δ and % Δ when available)	Adherence	Adverse effects
Alemo Munters et al. [45]	N(M/F): 21 (5/16) IG/CG: 11/10 Disease: PM/DM Mean age ± SD: 61.1 ± 15.2	57.14%; IG 1.25 (5), CG 2.50 (5)	Full	12-week AT at 50–70% VO_2_peak and RT at 30–40% of 1RM. 3x/week	Usual care	5RM, MMT-8, CPET, SF-36	↑ VO_2_peak (0.26; 17.44%), ↑ 5RM left knee extensor (2.7), ↔ 5RM right knee extensor (2.5; 33.1%), ↔ MMT-8 (1), ↑ SF-36 physical function (9.1), ↔ SF-36 general health (9.5), ↔ SF-36 mental health (5)	Not reported	None
Alemo Munters et al. [46]	N(M/F): 16 (2/14) IG/CG: 9/7 Disease: PM/DM Median age (range): 58 (37 to 77)	62.5%; IG 5 (1–8), CG (2–5)	Full	12-week AT at 50–70% VO_2_peak and RT at 30–40% of 1RM. 3x/week	Usual care	CPET, microdialysis, muscle biopsies	↑ TTE (17.5; 112.99%), ↓ lactate (− 5.2; − 51.03%), ↔ HRpeak (2; 2.06%), ↑ VO_2_peak (0.42; 26.77%), ↑ power at VO_2_peak (27; 23.24%), ↑ CS (36.4; 79.07%), ↑ β-HAD (23.7; 65.76%)	Not reported	None
Alexanderson et al. [47]	N(M/F): 19 (5/14) IG/CG: 10/9 Disease: PM/DM Median age (Q1-Q3): 60 (52–67)	100%; IG 9.5 (7.5–12.5), CG 10 (7.5–12.5)	Full	12-week AT (50–70% of HRpeak) and RT, 5x/week. Then, 12-week encouraged exercise	Range of motor exercises	FI, CPET	↔ FI, ↔ estimated VO₂	80%	Transitory muscle soreness
Ayyıldız et al. [48]	N(M/F): 66 (0/66) IG1/IG2/CG: 22/22/22 Disease: RA Mean age ± SD: 42.5 ± 5.6	69.70%; not reported	Partial	I1: 12-week RT 3x/week I2: 12-week AT at 85% of HRpeak 3x/week	Range of motion exercises	Ultrasound,DXA, 6MWT, TUG, 5-chair test, SF-36	↔ BMI, ↓ fat mass (− 0.98; − 3.17%), ↑ lean mass (0.51; 1.26%), ↔ 6MWD, ↓ TUG, ↔ 5-chair test, ↑ muscle thickness in biceps femoris and vastus intermedius (↔ in the rest of muscles), ↑ SF-36 pain (↔ rest of domains)	Not reported	None
Boehler et al. [49]	N(M/F): 13 (1/12) IG/CG: 6/7 Disease: PM/DM Mean age: 63	62.5%; not reported	Full	12-week AT at 50–70% VO_2_peak and RT 30–40% of 1RM. 3x/week	Usual care	Affymetrix microarray in muscle biopsies	MicroRNAs that reduce inflammation were upregulated and those inhibiting mitochondrial function were downregulated	Not reported	None
Bostrӧm et al. [50]	N(M/F): 35 (0/35) IG/CG: 18/17 Disease: SLE Mean age ± SD: 52.5 ± 9.4	51.43%; IG 3.1 (0–5), CG 1.3 (0–5)	Partial	I: 12-week AT and RT 2x/week, then 9 months self-managed	Usual care	CPET, SF-36	↔ VO_2_peak (0.06; 5.3%), ↓ SF-36 mental health (− 14; − 21.03%; ↔ rest of domains), ↔ prednisolone dose	80%	None
García et al. [51]	N(M/F): 60 (0/60) IG/CG: 30/30 Disease: Sjogren’s syndrome Mean age ± SD: 58.1 ± 11.5	81.67%; not reported	Full	16-weeks RT (3 × 12 at 80% MVC) and then 12-week AT at 20–84% of VO_2_peak. 2x/week	Usual care	CPET, blood analysis, Doppler echocardiography, SF-36	↑ VO_2_peak (5.07; 24.86%), ↔ LVEF, ↓ HbA1c, ↔ total cholesterol, ↔ HDL-C, ↔ LDL-C, ↔ triglycerides, ↔ SF-36	82%	Not reported
Hashemi et al. [52]	N(M/F): 19 (0/19) IG/CG: 10/9 Disease: SLE Mean age ± SD: 35.45 ± 10.74	63.16%; not reported	Full	8-week pilates and AT (50–60% VO_2_peak). 3x/week	Usual care	CPET, sit-ups, SLE questionnaire	↑ VO_2_peak (9.83; 28.78%)_,_ ↑ sit-ups (10.8; 64.98%), ↑ sit and reach (6.42; 18.18%), ↑ HRQOL (10.74; 14.13%)	Not reported	Not reported
Janse van Rensburg et al. [53]	N(M/F): 37 (0/37) IG/CG: 19/18 Disease: RA Mean age ± SD: 46.9 ± 8.1	54.04%; not reported	Full	12-week AT at 60–80% of HRpeak and RT. 2–3x/week	Usual care	Polar 810i strap	↑ RMSSD (5.3), ↑ pNN50 (− 0.2), ↔ LF, ↔ HF, ↔ LF/HF, ↑ SD1 (3.70), ↑ SD2 (6.80)	Not reported	Not reported
Miossi et al. [54]	N(M/F): 24 (0/24) IG/CG: 14/10 Disease: SLE Mean age ± SD: 31.2 ± 5.4	75%; not reported	Full	12-week AT and RT (4 × 8–12). 2x/week	Usual care	CPET	↑ Chronotropic reserve (14.6; 18%), ↑ HRR (15.5; 64.59%)	Not reported	None
Misra et al. [55]	N(M/F): 38 (22/16) IG/CG: 19/19 Disease: MG Mean age ± SD: 43.7 ± 13.8	100%; IG 15 (10–15), CG 15 (10–20)	None	12-week of AT for 30’/day	30-min rest daily	MMS, handgrip, MGADL, 6MWT, MG-QOL15	↑ MMS, ↔ handgrip strength (3.06; 13.15%), ↔ MGADL, ↑ 6MWD, ↑ MG-QOL15, ↓ prednisolone dose	97.3%	None
Naz et al. [56]	N(M/F): 18 (6/12) IG/CG: 9/9 Disease: sarcoidosis Median age: 59 in IG and 51 in CG	61.11%; IG 2 (0–5), CG 8 (0–24)	Partial	12-week RT and AT at 70–80% of 6MWT-derived capacity. 2x/week	Usual care	6MWT, leg dynamometer, SF-36, HADS	↑ 6MWD (60; 14.76%), ↑ leg strength (14; 25.07%), ↓ HADS anxiety (− 1; − 37.5%), ↓ HADS depression (− 2; − 40%), ↔ SF-36	100%	None
Rodrigues et al. [57]	N(M/F): 48 (0/48) IG1/IG2/CG: 16/16/16 Disease: RA Mean age ± SD: 58.6 ± 5.5	68.75%; not reported	Full	I1: 12-week RT at 70% 1RM 2x/week I2: 12-week blood-flow restriction at 30% 1RM 2x/week	Usual care	1RM, CT, TST, TUG, SF-36	↑ 1RM leg press (28.73; 25.3%), ↑ 1RM knee extension (8.24; 23.4%), ↑ quadriceps CSA (9.9%), ↑ TST (15.3%), ↓ TUG (− 8.4%), ↑ SF-36 physical function (7.72%) and pain (4.74%, ↔ rest of domains)	86.6%	One case of exercise-induced patellofemoral pain
Shapoorabadi et al. [58]	N(I/C): 33 (0/33) IG/CG: 16/17 Disease: sarcoidosis Mean age ± SD: 52 ± 7.8	100%; mean for all participants 5 mg/day	Full	I: 8-week AT at 60–70% of HRR, 3x/week	Usual care	Blood analysis	↓ RBC (− 0.25; − 5.16%), ↑ Hb (1.02; 9.63%), ↑ HCT (2.43; 6.91%)	Not reported	Not reported
Siqueira et al. [59]	N(M/F): 100 (0/100) IG1/IG2/CG: 33/33/34 Disease: RA Mean age ± SD: 54.1 ± 6.9	50.75%; not reported	Full	I1: 16-week land-based AT at 5–8 Borg CR-10. 3x/week I2: 16-week water-based AT at 5–8 Borg CR-10. 3x/week	Usual care	Isokinetic dynamometer, HAQ-M, DXA	↓ HAQ-M (− 0.4; − 48.25%), ↔ muscle strength (− 0.7; − 0.86%), ↔ weight (− 0.1; − 0.16%), ↔ BMI (1.2; 4.31%), ↔ body fat percentage (− 0.1; − 0.19%), ↔ bone mineral content (0.01; 0.34%), ↔ BMD (− 0.03; − 0.86%), ↔ lean mass (0.3; 0.87%)	Not reported	None
Strasser et al. [60]	N(M/F): 40 (4/36) IG/CG: 20/20 Disease: RA Mean age ± SD: 57.5 ± 8.9	50%; range 2.5 to 7.5 mg/day	Full	6-month RT (10–15 repetitions at 70% of 1RM) and AT (60% of VO_2_peak). 2x/week	Stretching exercises	HAQ-DI, CPET, 1RM, anthropometry	↔ HAQ-DI, ↔ W_max_ (10.64; 11.09%)_,_ ↔ 1RM leg press (14.47, 21.69%), ↔ 1RM bench press (4.13, 14.45%), ↔ 1RM bench pull (2.2, 7.42%), ↔ body weight (− 1.14, − 1.66%), ↔ body fat percentage (− 4.43; − 12.86%), ↔ lean body mass (2.93, 6.21%)	75%	Not reported
Tiffreau et al. [61]	N(M/F): 21 (7/14) IG/CG: 10/11 Disease: PM Mean age ± SD: 54.9 ± 13.1	100%; range 5–10 mg/day	Partial	4-week RT at 60% of 1RM and AT at 60% of HRpeak, 3x/week. Then 11-month daily 30-min sessions	Physiotherapy	HAQ-DI, MFM, 6MWT, Kendall MMT, isokinetic assessments, SF-36	↓ HAQ-DI (− 0.58, − 47.54%), ↔ MFM, ↑ Kendall MMT left side (9.27, 12.37%), ↔ Kendall MMT right side, ↔ peak isokinetic torque, ↔ 6MWD, ↑ SF-36 physical function (37.35, 91.91%) and general health (14.74, 35.52%; ↔ rest of domains)	Not reported	Not reported
Wallaert et al. [62]	N(M/F): 38 (17/21) IG/CG: 20/18 Disease: sarcoidosis Median age (IQR): 57.5 (48—65)	55.26%; 5(0–15)	Full	2-month RT and AT, 3x/week	Oral counselling	6MST, VSRQ, HADS	↑ 6MST, ↔ anxiety, ↔ depression, ↔ VSRQ, ↔ prednisone dose	18/20 participants completed all the sessions	None
Westby et al. [63]	N(M/F): 30 (0/30) IG/CG: 14/16 Disease: RA Mean age ± SD: 56.2 ± 10.3	100%; 4.99 (2.11)	None	12-month AT + RT, 3x/week. Low-moderate intensity	Written information	DPX, HAQ-DI	↔ BMD (0.027; 2.72%), ↔ HAQ-DI (0)	71%	Not reported

↑ significant increase in intervention group compared to control group, ↓ significant decrease in intervention group compared to control group, ↔ no significant between-group differences, *6MST* 6-min stepper test, *6MWD* 6-min walking distance, *6MWT* 6-min walking time test, *β-HAD* β-hydroxyacyl-CoA dehydrogenase, *AT* aerobic training, *BMD* bone mineral density, *BMI* body mass index, *CG* control group, *CPET* cardiopulmonary exercise testing, *CR-10* category-ratio 10 scale, *CS* citrate synthase, *CSA* cross-sectional area, *CT* computed tomography imaging, *DM* dermatomyositis, *DPX* dual energy projection radiology, *DXA* dual-energy X-ray absorptiometry, *F* female subjects, *FI* Functional Index, *GC* glucocorticoid, *HADS* Hospital Anxiety and Depression Scale, *HAQ-DI* health assessment questionnaire–disability index, *HAQ-M* health assessment questionnaire–modified, *Hb* hemoglobin, *HbA1c* glycated hemoglobin, *HCT* hematocrit, *HDL-C* high-density lipoprotein cholesterol, *HF* high frequency, *HRpeak* peak heart rate, *HRQOL* health-related quality of life, *HRR* heart-rate reserve, *HRV* heart rate variability, *IG* intervention group, *LDL-C* low-density lipoprotein cholesterol, *LF* low frequency, *LVEF* left ventricular ejection fraction, *M* male subjects, *MFM* motor function measure, *MG* myasthenia gravis, *MGADL* myasthenia gravis activities of daily living, *MG-QOL15* myasthenia gravis quality of life 15-item scale, *MMS* myasthenic muscle score, *MMT* manual muscle strength testing, *MVC* maximum voluntary contraction, *n* number of participants, *PM* polymyositis, *pNN50* percentage of successive normal-to-normal intervals differing by more than 50 ms, *RA* rheumatoid arthritis, *RBC* red blood cell, *RM* repetition maximum, *RMSSD* root mean square of successive differences, *RT* resistance training, *SD* standard deviation, *SF* Short-Form-36 test, *SLE* systemic lupus erythematosus, *TST* timed-stands test, *TTE* time to exhaustion, *TUG* Timed Up and Go, *VO*_*2*_*peak* peak oxygen consumption, *VSRQ* visual simplified respiratory questionnaire, *W*_*max*_ maximum workload in watts

### Intervention Characteristics

Exercise interventions were predominantly supervised and typically incorporated combined aerobic and resistance training modalities. Program durations most commonly spanned 12 weeks, with sessions conducted two to three times per week. Progressive moderate-intensity protocols were most frequently implemented.

Control groups were generally assigned to usual care or low-intensity interventions, including stretching, range of motion exercises [46, 50, 55], or physiotherapy [64]. Several studies also incorporated verbal counselling. Most protocols adhered to progressive overload principles, although details regarding progression schemes and adherence rates were reported inconsistently across studies.

### Outcomes

Where available, we report absolute (Δ) and relative (%Δ) changes and compare them with established minimally clinically important differences (MCIDs) or pragmatic thresholds from similar populations: 6MWD (~ 14–30 m), TUG (~ 0.8–1.4 s), gait speed (0.05 m·s⁻^1^ small; 0.10 m·s⁻^1^ substantial), VO_2_peak (~ 3.5 mL·kg⁻^1^·min⁻^1^ ≈ 1 MET), handgrip (~ 5–6% in older adults), 36-item Short Form Health Survey (SF-36, ≈3–5 points per domain), and glycated hemoglobin (HbA1c, ≥ 0.3–0.5% absolute reduction) [65–71]. These comparators aid in interpreting trial findings considering disease heterogeneity.

#### Muscle Function and Structure

Muscle strength was assessed in the majority of included studies using various methodological approaches. Two studies employed one-repetition maximum (1RM) testing: Rodrigues et al. [57] reported significant gains in both leg press and knee extension strength, whereas Strasser et al. [60] found no significant changes across multiple exercises, including leg press, bench press, and bench pull. Using five-repetition maximum (5RM) testing, Alemo Munters et al. [45]*,* noted improvement in left knee extensor strength but not in the right.

Isokinetic and isometric dynamometry yielded inconsistent findings. While Siqueira et al. [59] reported significant strength improvements, Naz et al. [56] observed no meaningful changes in strength parameters. Similarly, Misra et al. [55] found no significant improvement in handgrip strength. Results from manual muscle testing (MMT) were equally heterogeneous, with some studies reporting benefits [61] and others showing no change [45].

At the molecular level, two studies explored exercise-induced muscle adaptations. Boehler et al. [49] demonstrated that training modulated 39 muscle microRNAs (miRNAs), upregulating anti-inflammatory miRNAs while downregulating those involved in mitochondrial suppression. Alemo Munters et al. [46] reported significant increases in mitochondrial enzyme activity, including citrate synthase (CS) and β-hydroxyacyl-CoA dehydrogenase (β-HAD), indicating enhanced muscle oxidative capacity.

Muscle morphology outcomes demonstrated greater consistency. Significant increases in muscle thickness (assessed via ultrasound) [48] and quadriceps cross-sectional area (CSA) (via computed tomography) [57] were observed following exercise interventions.

Across strength outcomes, studies reporting raw values typically showed Δ1RM leg press in the range of 25–28 kg (%Δ ~ 23.5%) and knee extension 5–8 kg (%Δ ~ 21.75%) [57]. In older adults and inflammatory myopathies, ~ 5–10% strength gain is commonly regarded as functionally meaningful; gains ≥ 15% align with improvements in chair-rise and stair-climb performance. Ultrasound/computed tomography showed vastus lateralis increases of 400–500 mm^2^ (%Δ ~ 10%), consistent with hypertrophic adaptation and typically sufficient to support the observed strength gains [57]. Mitochondrial enzyme increases (e.g., CS, β-HAD) of 50–79% indicate improved oxidative capacity, a prerequisite for higher fatigue resistance [46].

#### Body Composition

Body composition was evaluated in two studies with divergent findings. Ayyildiz et al. [48] demonstrated significant reductions in fat mass and increases in lean mass following PRT. In contrast, Strasser et al. [60] detected no substantial changes in fat or lean tissue composition. Only one study [63] examined BMD and revealed no between-group differences.

Trials reporting dual-energy X-ray absorptiometry (DXA) showed for fat mass a reduction of 0.48 kg (%Δ ~ 1.6%) and lean mass an increase of 1.11 kg (%Δ ~ 2.8%) [48]. In the obesity literature, ~ 2–3% total fat reduction or ≥ 0.5–1.0 kg lean mass gain over 12 weeks is typically considered meaningful, especially when accompanied by strength or functional improvements.

#### Physical Function

Several trials evaluated functional outcomes using validated performance assessments. Significant improvements were observed in the TUG test following exercise interventions across multiple studies [48, 57]. Similarly, the 6MWD demonstrated benefits in two RCTs [55, 56], although two other studies reported null findings [48, 61]. Results for VO_2_peak were similarly mixed: Alemo Munters et al. [46] reported significant gains, whereas Boström et al. [50] found no effect.

Functional tests improved by Δ6MWD ~ 40 m (often exceeding the MCID of 14–30 m), and by ΔTUG ~ 1–1.25 s (approaching/exceeding the 0.8–1.4 s MCID) [56, 57]. When VO_2_peak (mL·kg⁻^1^·min⁻^1^) changed, Δ ~ 24% approximated a magnitude linked to 13–15% reductions in cardiovascular and all-cause mortality in large cohorts [68].

#### Health-Related Quality of Life and Mental Health

Seven studies assessed the health-related quality of life using the SF-36. Among these, only one study reported significant post-intervention improvement [50]. Two RCTs evaluated anxiety and depression using the Hospital Anxiety and Depression Scale (HADS) [56, 62], with only one trial documenting significant reductions in anxiety symptoms [56], while the other reported no effect.

Where SF-36 was used, domain changes seldom exceeded the 3- to 5-point MCID, which may explain inconsistent significance. Anxiety reductions (HADS-A Δ ~ 2) met the ~ 1.5- to 1.7-point responder threshold in the positive trial, aligning with effects seen in supervised exercise for mood disorders.

#### Cardiovascular Risk Factors

Direct measures of cardiovascular risk (e.g., incidence of cardiovascular events) were not assessed. However, indirect markers were evaluated in selected studies. Garcia et al. [51] reported stable left ventricular ejection fraction (LVEF) post-exercise, while Janse van Rensburg et al. [53] demonstrated enhanced cardiac autonomic function. Additionally, Garcia et al. [51] reported significant reductions in HbA1c levels following the intervention (Table 3).

**Table 3:** Evidence-to-recommendation framework incorporating RoB2 risk-of-bias assessment

Outcome domain	Exercise intervention effect		Recommendations	
	Direction of effect	Evidence confidence (RoB2 + number of studies)	Directness (CG trials vs. extrapolated)	Key implementation notes
Muscle strength	Beneficial effect	Moderate	Direct GC trials	Whole-body progressive resistance training 2–3x/wk
Physical function	Beneficial effect	Moderate	Direct GC trials	
Body composition	Mixed evidence	Low	Direct GC trials	≥ 150 min/week moderate- or ≥ 60 min/week vigorous-intensity activity
Cardiometabolic markers	Mixed evidence	Low	Extrapolated from diabetes mellitus and hypertension trials	Daily aerobic exercise at moderate intensity combined with ≥ 2 × /week resistance training Extended cooldowns and proper breathing Monitor BP Emphasize post-meal exercise timing
Bone	Uncertain	Very low	Extrapolated from osteoporosis trials	Emphasis on spinal extensor strength Spine-sparing techniques
Mental health	Mixed evidence	Low	Extrapolated from depression and anxiety trials	Adapt to individual preferences Prioritize supervised, group-based, and recreational settings

*BP* blood pressure, *GC* glucocorticoids, *x/week* times per week

In the study reporting glycemia, HbA1c decreased by Δ ~ 0.13%, which even if statistically significant, did not meet the ≥ 0.3–0.5% threshold commonly viewed as clinically relevant in cardiometabolic care. Improvements in cardiac autonomic function (e.g., heart rate-variability indices) are consistent with the known links between supervised training and lower sympathetic tone, a mechanism connected to blood-pressure and fatigue improvements.

#### Medication Dosage

Three studies examined changes in glucocorticoid dosage. Misra et al. [55] reported significant reductions in prednisolone dose following the intervention. In contrast, two other studies [50, 62] observed no significant differences between exercise and control groups.

Where prednisolone dose decreased (Δ ~ 1.26 mg·day⁻^1^), this represented a drug-sparing signal. Even modest reductions (e.g., 2.5–5 mg·day⁻^1^) sustained over months are associated with lower fracture, diabetes, and infection risk in pharmaco-epidemiology.

#### Summary

The studies included in this systematic review section demonstrated significant molecular and morphological muscle adaptations following exercise interventions. Additionally, exercise interventions resulted in improvements in physical function, body composition, and cardiac autonomic function. However, most studies found no significant benefits in health-related quality of life, which may be attributed to the use of generic instruments, short intervention duration, and limited power to detect change. Due to the limited number of included studies and their contradictory results, findings regarding glucose metabolism and medication dosage remain inconclusive.

Overall, several trials achieved changes at or above accepted MCIDs for 6MWD, TUG, strength, and body composition, indicating clinically meaningful benefits beyond statistical significance. Heterogeneity and small samples likely explain null results in HbA1c and generic health-related quality of life (HRQoL), outcomes that usually require longer duration or higher training dose.

Evidence confidence was moderate for improvements in muscle-related outcomes and certain functional tests, but low/very low for bone, cardiometabolic, and mental health outcomes due to the small number of studies, risk of bias in the included studies, and heterogeneity in outcome assessment.

Reporting of adverse events was inconsistent across trials; where reported, supervised programs were generally well tolerated, but the absence of standardized harms reporting limits conclusions regarding fracture- or cardiovascular-related safety in high-risk subgroups.

## Recommendations for Tailoring Physical Exercise Prescription to Glucocorticoid Treatment

To support bedside implementation, we propose a stepwise assessment-to-prescription pathway (ESM Fig. S3) integrating complication-specific risk stratification with frequency, intensity, time, type, volume and progression (FITT-VP) exercise parameters and safety monitoring.

Additionally, the recommendations below are informed by (i) trials in glucocorticoid-treated populations identified in this review (direct evidence) and (ii) high-quality guidelines for osteoporosis, hypertension, diabetes, and mental health where glucocorticoid-specific exercise trials are lacking (indirect evidence).

### Bone Health

#### Indirect Evidence: Specific Guidelines for Osteoporosis

International guidelines recommend PRT and balance training as fundamental components of exercise regimens for individuals with osteoporosis or those at risk of glucocorticoid-induced bone loss [64, 72]. Resistance training should be performed two to three times per week, with particular emphasis on the spinal extensor muscles, which are crucial for posture and vertebral fracture prevention [64, 72, 73]. To minimize fracture risk, safe movement strategies must be employed, including “spine-sparing techniques” that avoid high-risk actions such as deep forward spinal flexion [64, 73]. The incorporation of impact exercises (e.g., jumping and brisk walking) should be individualized based on careful assessment of pain levels, joint integrity, and overall fracture risk [72].

### Hypertension

#### Indirect Evidence: Specific Guidelines for Hypertension

Exercise prescriptions for individuals with glucocorticoid-induced or pre-existing hypertension should incorporate both aerobic and resistance training modalities to optimize cardiovascular benefits [25]. Aerobic training at moderate intensity (40–60% VO_2_peak) for at least 30 min daily is recommended to sustain post-exercise hypotensive effects [74]. Progressive resistance training, performed at 40–60% of 1RM two or more times per week, also provides significant antihypertensive benefits [74, 75]. Exercise programs should incorporate 8–10 exercises targeting major muscle groups, with two to three sets of 8–12 repetitions for each exercise [75].

Patients must be instructed to practice controlled breathing during resistance exercises to avoid the Valsalva maneuver, which may acutely elevate blood pressure [76]. Each exercise session should conclude with an extended cool-down phase to prevent abrupt hemodynamic changes [77]. Clinicians should maintain vigilant monitoring for cardiovascular warning signs (e.g., chest pain, abnormal dyspnea, and dizziness) and conduct regular assessments of both resting and exercise blood pressure [76, 77].

### Weight Gain

#### Direct Evidence: RCTs in Glucocorticoid-Treated Populations

Glucocorticoid-related weight gain and central adiposity mainly result from changes in energy balance and substrate distribution. Therefore, although exercise provides additional metabolic benefits and helps sustain long-term weight loss, dietary restriction remains the main factor for short-term weight loss. A practical strategy combines a slightly hypocaloric plan (around 300–500 kcal·day⁻^1^ deficit) with sufficient protein intake (1.2–1.6 g·kg⁻^1^·day⁻^1^) to maintain fat-free mass during weight loss, preferably under a dietitian’s supervision [78].

Aerobic exercise should be prescribed at a dose sufficient to influence energy balance and visceral fat. For most patients, this corresponds to ≥ 150–300 min·week⁻^1^ of moderate-intensity exercise (40–60% of VO₂ reserve (VO₂R)/heart-rate reserve (HRR); rate of perceived exertion (RPE) 12–13) or ≥ 75–150 min·week⁻^1^ of vigorous-intensity exercise (≥ 60–85% VO₂R/HRR; RPE 14–17) [78, 79]. When appropriate clinically, high-intensity interval training (HIIT)—such as 4–10 intervals of 1–4 min at 85–95% of peak heart rate (HR_peak_) with equal or longer recovery, performed two to three times per week—can yield similar or greater reductions in total and visceral fat than moderate-intensity continuous training, while offering comparable cardiometabolic benefits [78, 79]. Given the high prevalence of hypertension and autonomic dysregulation in populations treated with glucocorticoids, HIIT should be introduced gradually with close blood pressure monitoring and extended cool-downs (see Sect. 4.2). For weight-loss maintenance, patients should progress toward the upper range of 200–300 min·week⁻^1^ of aerobic activity, supported by ambulatory goals (e.g., > 7,000–8,000 steps·day⁻^1^) and ongoing PRT [78, 79].

Supervised combined training programs that incorporate both aerobic and resistance exercise modalities are strongly recommended to optimize outcomes [79]. To enhance long-term adherence, implementation strategies should prioritize fostering patient autonomy, avoiding stigmatizing language, and establishing realistic expectations that emphasize overall health improvements and functional capacity rather than focusing exclusively on weight loss [78].

### Muscle Function

#### Direct Evidence: RCTs in Glucocorticoid-Treated Populations

Progressive resistance training represents the first-line intervention for managing glucocorticoid-induced myopathy [80]. Training protocols should employ whole-body approaches that target major muscle groups while incorporating functional balance exercises to mitigate fall risk and enhance postural control [81]. Programs must adhere to principles of progressive overload, with systematic adjustments to intensity and volume based on individual capacity and recovery patterns [80]. When 1RM testing is not feasible, intensity can be prescribed using RPE (e.g., RPE 5–7/10) or repetitions-in-reserve (leave ~ 2–4 repetitions in reserve), progressing volume and load as tolerated. Given the elevated risk of sarcopenia and muscle weakness in this population, early intervention initiation and regular monitoring of muscle strength and functional mobility are essential components of comprehensive care.

### Hyperglycemia

#### Indirect Evidence: Specific Guidelines for Diabetes Mellitus

In accordance with established clinical guidelines for type 2 diabetes prevention and management, adults—particularly those with glucocorticoid-induced insulin resistance—should engage in at least 150 min per week of moderate-to-vigorous aerobic exercise, distributed across a minimum of 3 days with no more than two consecutive days of inactivity [82, 83].

Combined aerobic and resistance training protocols demonstrate superior glycemic control compared with either modality implemented independently [82]. Current evidence supports integrating both exercise modalities within individual sessions, with resistance training optimally performed before aerobic exercise to maximize metabolic benefits [84].

Resistance-training components should target major muscle groups through eight to ten exercises performed in one to three sets per muscle group, conducted on at least two non-consecutive days per week [82, 84]. Gradual progression in both intensity and volume is essential for promoting physiological adaptation while minimizing injury risk [82]. Exercise timing considerations are also clinically relevant, as postprandial exercise may enhance post-meal glucose regulation [82]. Supervised programs demonstrate superior efficacy compared with unsupervised approaches and should be individualized based on patient preferences, motivational factors, and contextual circumstances [82, 83].

Beyond structured training interventions, reducing sedentary behavior through strategies such as incorporating frequent light-intensity movement breaks can significantly improve glycemic responses and attenuate postprandial glucose excursions [82, 83].

### Cognitive Function

#### Indirect Evidence: Specific Guidelines for Cognitive Function

Emerging evidence demonstrates that multicomponent exercise interventions performed five to seven times per week can significantly enhance cognitive function in individuals at risk of glucocorticoid-related cognitive impairment [85]. These comprehensive programs should incorporate a minimum dose of resistance training (30–60 min, at least twice weekly) to optimize neurocognitive benefits [86].

To facilitate compliance and adaptation, machine-based resistance exercises targeting major muscle groups are recommended, particularly for individuals with cognitive limitations. These modalities allow for controlled intensity progression while reducing cognitive load demands [86]. Power-training protocols that incorporate cognitive components, such as reaction time tasks, have demonstrated efficacy in improving both cognitive and physical outcomes simultaneously [87].

Furthermore, the integration of motivational exergames—which combine physical activity with game-like features—may enhance both neurocognitive benefits and adherence to long-term training regimens, offering a promising approach for sustained engagement [86].

### Mental Health

#### Indirect Evidence: Specific Guidelines for Depression and Anxiety

A diverse array of exercise modalities—including yoga, walking, and HIIT—have demonstrated efficacy in reducing depressive symptoms, particularly when delivered in socially supportive environments [88]. Accordingly, supervised and group-based formats tailored to individual patient preferences are recommended to enhance engagement and promote long-term sustainability [89].

For individuals experiencing anxiety symptoms, high-intensity exercise may confer greater therapeutic benefits compared to low-intensity exercise, although the current evidence base remains limited and warrants further investigation [90]. Across all interventions, fostering an environment that supports the fundamental psychological needs of autonomy, competence, and relatedness is critical for building intrinsic motivation and promoting sustained participation in exercise programs [91].

### Summary

**Fig. 3:**
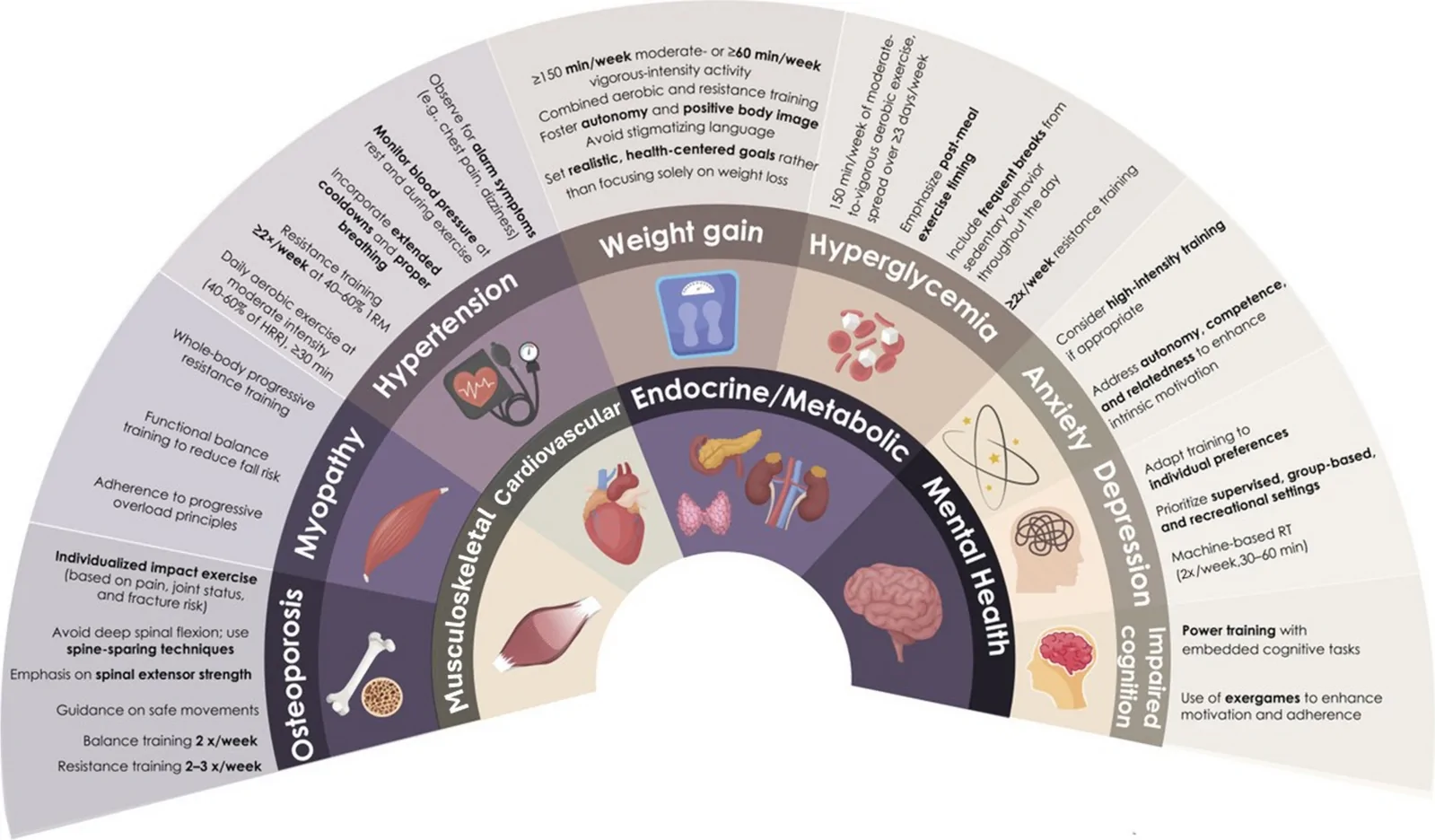
Exercise recommendations for the management of glucocorticoid-induced adverse effects. *1RM* one-repetition maximum, *HRR* heart-rate reserve, *RT* resistance training, *x/week* times per week

When oral long-term glucocorticoids are prescribed, tailored exercise recommendations should be implemented to mitigate medication-related adverse effects while addressing patients’ individual clinical profiles and disease-specific needs (ESM Table S2 and Fig. 3).

Combined resistance and aerobic training remain the cornerstones of therapeutic exercise prescription for glucocorticoid-treated patients. Resistance training should target major muscle groups and be performed two to three times per week at 40–60% of 1RM. To further support musculoskeletal and cognitive resilience, protocols may incorporate muscle power exercises combined with cognitive tasks, particularly for individuals at risk of cognitive impairment. The inclusion of functional balance exercises is critical for reducing fall risk and countering the musculoskeletal effects of glucocorticoid-induced sarcopenia and osteoporosis.

Aerobic training should be prescribed at least three times per week, performed at moderate intensity (40–60% of HRR), and adjusted progressively according to the patient’s tolerance and fitness level. Both aerobic and resistance training programs must adhere to the principles of progressive overload to optimize physiological adaptations while minimizing injury risk.

Group-based supervised interventions are strongly recommended to ensure safety, maintain technique fidelity, and provide essential psychosocial support. To promote long-term adherence, exercise programs should be tailored to individual preferences, emphasizing patient autonomy and competence while aligning with each person’s functional goals and health priorities. Setting realistic expectations—with a focus on holistic health outcomes rather than symptom control alone—is essential for sustaining motivation and therapeutic engagement in glucocorticoid-treated patients.

Safety monitoring is critical when prescribing exercise to patients receiving glucocorticoids, particularly those with osteoporosis, hypertension, or sarcopenia. Clinicians should conduct comprehensive baseline assessments of musculoskeletal integrity, cardiovascular status, and glycemic control, adjusting protocols accordingly. The emergence of adverse symptoms during exercise (e.g., dizziness, abnormal fatigue, or musculoskeletal pain) should prompt immediate reassessment of training intensity and program structure. Additionally, the search strategy used to identify recommendations for exercise prescription is detailed in ESM Methods S3.

## Limitations and Strengths of the Review

This narrative review, which includes a systematic review section, possesses several notable strengths that enhance its contribution to the literature. Most importantly, it provides a comprehensive synthesis of available evidence examining the interactions between glucocorticoid therapy and physical exercise—an underexplored yet clinically critical domain. The review uniquely bridges pharmacological and exercise science perspectives by proposing tailored, evidence-based recommendations that address specific glucocorticoid-induced complications, an approach rarely integrated in current clinical guidelines.

Nevertheless, several important limitations must be acknowledged. First, substantial heterogeneity existed among the included RCTs regarding intervention design, study populations, and outcome measures. This heterogeneity significantly limited direct comparability between studies and precluded meaningful quantitative synthesis through meta-analysis. Second, a notable paucity of high-quality studies specifically assessed exercise effects on key glucocorticoid-induced complications, particularly bone health, cardiometabolic outcomes, and neurocognitive function. Third, most included trials were conducted in females with rheumatic diseases, limiting generalizability to other high-prevalence glucocorticoid indications (e.g., asthma/COPD) and to male patients. Therefore, while recommendations were systematically structured around established glucocorticoid-related adverse effects, the populations examined in most included trials may not fully represent the broader clinical spectrum of patients who typically receive long-term glucocorticoid therapy in real-world practice.

Despite encouraging evidence supporting exercise interventions for muscle-related outcomes, substantial research gaps persist that warrant urgent attention. Notably, few trials have examined BMD, insulin sensitivity, or fall and fracture risk as primary endpoints, representing critical knowledge deficits given the well-established skeletal and metabolic complications of chronic glucocorticoid use. Furthermore, no identified study has directly compared exercise efficacy across different glucocorticoid dosages, treatment durations, or delivery routes—factors that likely influence both the magnitude of adverse effects and the potential for exercise-mediated mitigation. Moving forward, there exists a pressing need for well-designed mechanistic trials that elucidate the biological pathways underlying exercise-glucocorticoid interactions, as well as longitudinal real-world studies that can inform practical implementation strategies across diverse clinical populations.

## Conclusions

This narrative review, which includes a systematic review section, provides a comprehensive analysis of the complex interactions between glucocorticoid therapy and physical exercise, with particular emphasis on optimizing clinical care for patients requiring long-term glucocorticoid treatment. The multisystem adverse effects of glucocorticoids—including diabetes mellitus, musculoskeletal disorders, hypertension, weight gain, cognitive impairment, and neuropsychiatric complications—create substantial barriers to both physiological exercise responses and long-term adherence to physical activity interventions.

Current evidence from RCTs demonstrates that structured exercise interventions can yield meaningful improvements in muscle strength, skeletal muscle architecture, and selected measures of physical function in glucocorticoid-treated patients. However, consistent therapeutic benefits across broader clinical domains—including quality of life, mood disorders, and metabolic control—remain elusive, likely reflecting methodological limitations, inadequate statistical power, or insufficient intervention duration in existing studies. Critical knowledge gaps persist regarding exercise effects on clinically relevant endpoints such as fracture prevention, glycemic control, blood pressure regulation, and cognitive preservation.

Future research priorities should emphasize high-quality, adequately powered clinical trials that stratify participants by glucocorticoid exposure duration and cumulative dose, incorporate clinically meaningful primary endpoints, and evaluate long-term outcomes through extended follow-up periods. Exercise prescriptions should be individualized and evidence-based, incorporating progressive resistance training and moderate-intensity aerobic exercise delivered under professional supervision, with integrated adherence-enhancement strategies to address the unique challenges faced by this patient population.

Successful clinical implementation of exercise interventions in glucocorticoid-treated patients necessitates coordinated interdisciplinary care involving rheumatologists, endocrinologists, physical therapists, and certified exercise specialists. Integration of exercise programs into established rehabilitation pathways, utilization of telehealth exercise platforms, and implementation of structured behavioral support systems represent promising strategies to enhance adherence, particularly among patients with mobility limitations or cognitive impairment.

The integration of exercise as an adjuvant therapeutic modality in glucocorticoid pharmacotherapy represents a paradigm shift with substantial potential to mitigate treatment-related adverse effects, reduce polypharmacy burden, and promote sustainable lifestyle modifications. Clinicians should treat glucocorticoid therapy as a modifier of exercise risk/benefit and prescribe exercise with the same intentionality as dose titration. By bridging the traditional divide between pharmacological and behavioral interventions, this approach may fundamentally improve long-term clinical outcomes while advancing toward a more comprehensive, patient-centered model of chronic disease management that addresses both the benefits and consequences of essential glucocorticoid therapy.

## Supplementary Information

Below is the link to the electronic supplementary material.

Supplementary file1 (PDF 751 KB)

## Declarations

### Data availability statement

Data corresponding to this review will be shared upon reasonable request to the corresponding author.
