# Supplementary material for: Physical Exercise as an Adjuvant to Glucocorticoid Therapy: Clinical Recommendations and Evidence Review
Source: Sports Med. 2026 Jun 8;56(10):2435–55. doi: 10.1007/s40279-026-02469-6 (PMC13614985; doi:10.1007/s40279-026-02469-6)
Supplement: Supplementary file 1 [file 40279_2026_2469_MOESM1_ESM.pdf]

Supplementary material

Table of Contents

1. Supplementary Figure S1. Risk of bias in each trial.
2. Supplementary Figure S2. Risk of bias graph.
3. Supplementary Figure S3. Clinical pathway for exercise prescription during systemic glucocorticoid therapy.
4. Supplementary Table S1. Glucocorticoid-induced adverse effects and their interactions with exercise capacity, engagement, and adaptation.
5. Supplementary Table S2. Tailored exercise recommendations for managing glucocorticoid-induced adverse effects.
6. Supplementary Methods S1. Effects of glucocorticoids on exercise capacity, engagement, and physiological adaptations.
7. Supplementary Methods S2. Systematic review of RCTs assessing exercise interventions in adults taking oral glucocorticoids.
8. Supplementary Methods S3. Exercise recommendations.
9. References.

1. Supplementary Figure S2. Risk of bias in each trial.

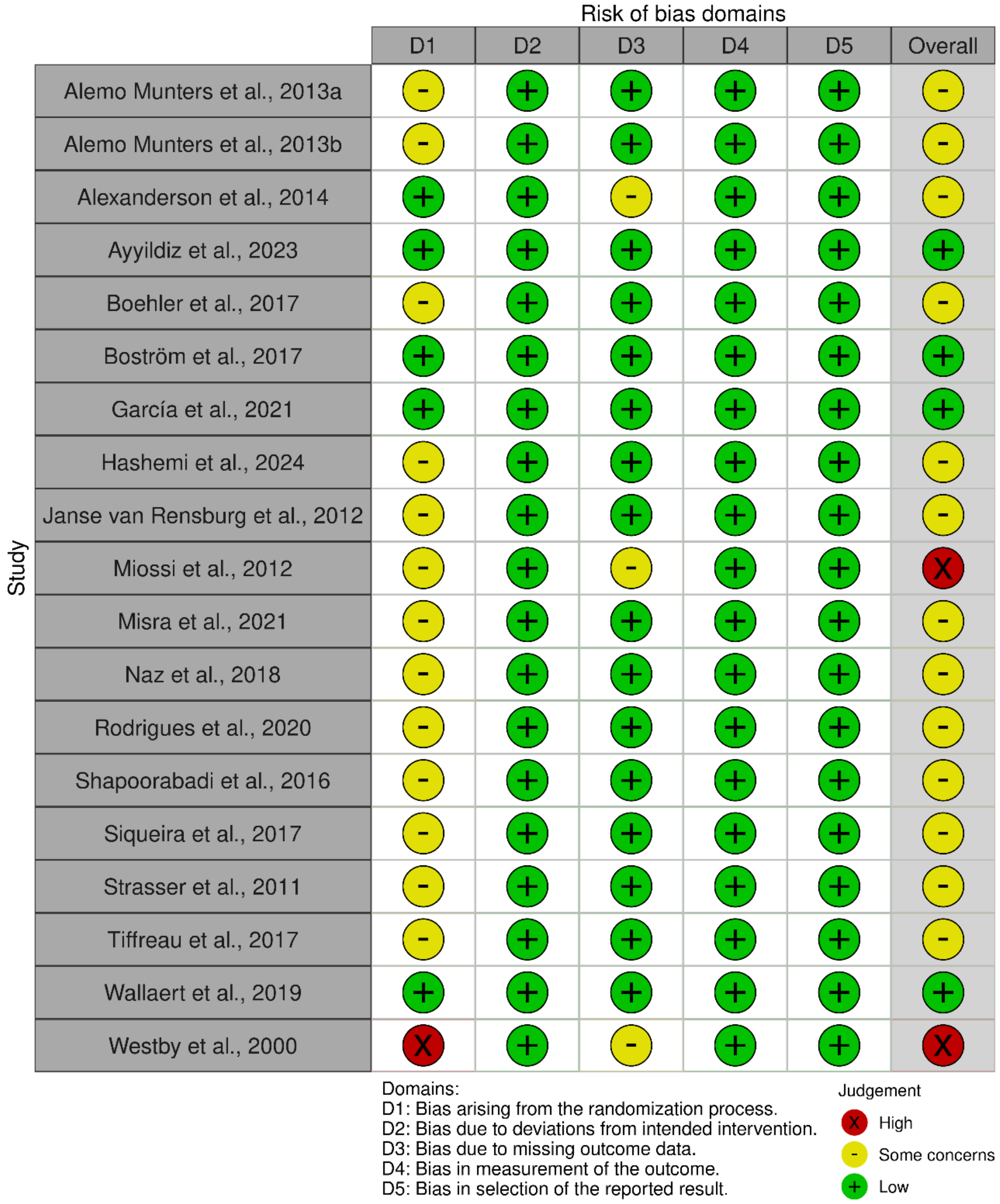

2. Supplementary Figure S3. Risk of bias graph.

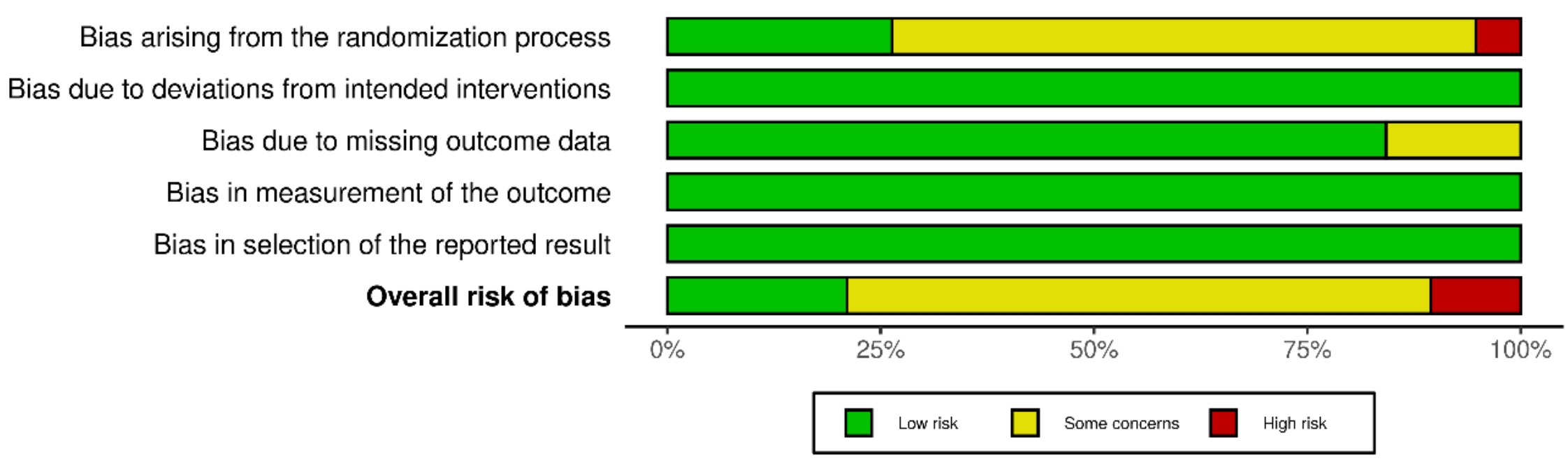

3. Supplementary Figure S3. Clinical pathway for exercise prescription during systemic glucocorticoid therapy.

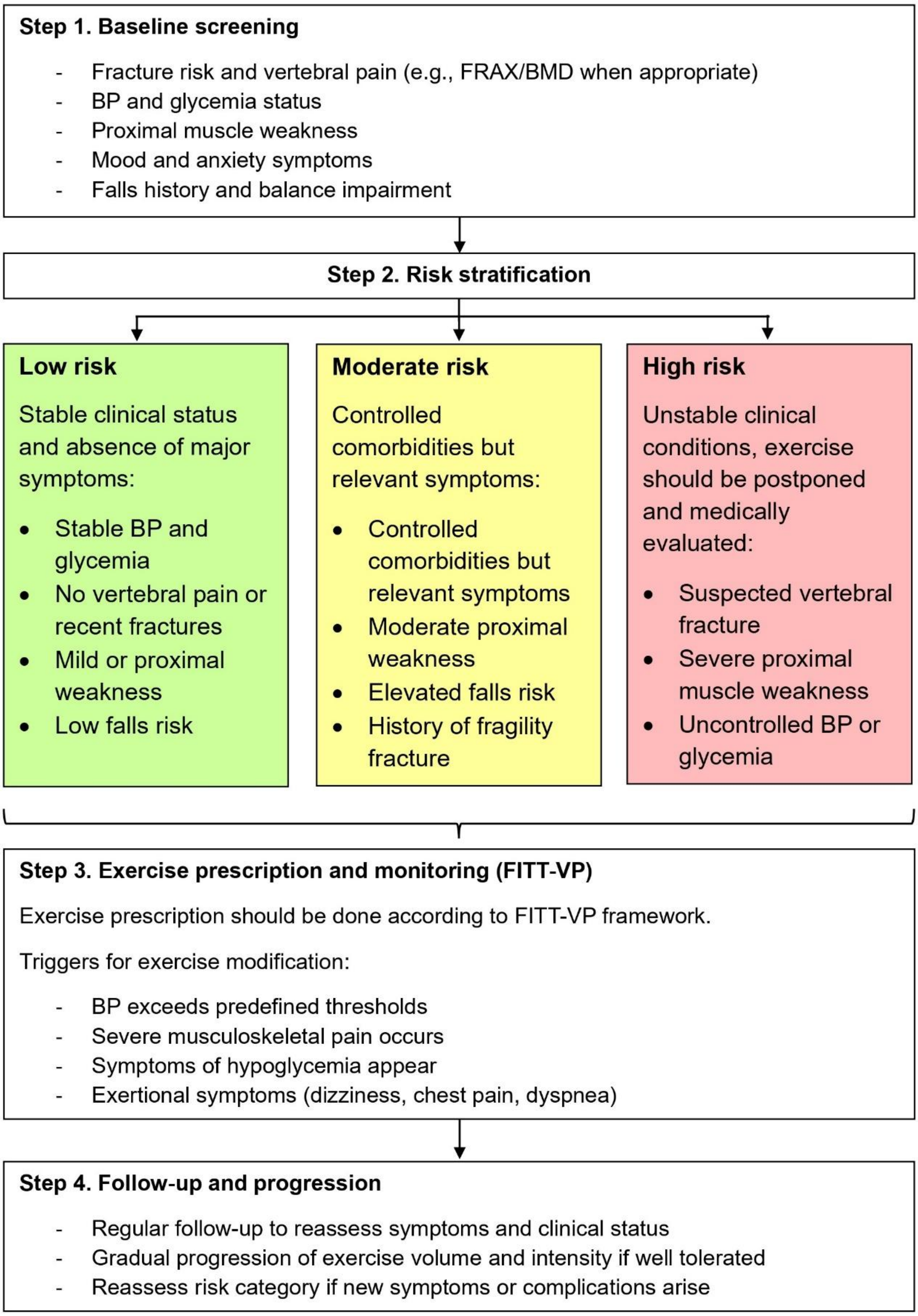

4. Supplementary Table S1. Glucocorticoid-induced adverse effects and their interactions with exercise capacity, engagement, and adaptation.

Interaction type	Adverse effect	Impact on Exercise
Glucocorticoid-exercise capacity interactions	Osteoporosis	• Increased **risk of fragility fractures.**<br>• **Balance deficits** due to vertebral deformity or pain.<br>• Reduced **mobility** and confidence in movement.
	Osteonecrosis	• **Pain-induced movement limitation.**<br>• **Risk of joint collapse** under load-bearing activities.
	Hypertension	• **Reduced cardiovascular tolerance**.<br>• Need for **blood pressure monitoring** before, during, and after sessions.<br>• Elevated **CV risk.**
	Impaired cognitive function	• **Reduced executive function**, memory, and attention.<br>• Challenges in **following instructions** or complex routines.<br>• Risk of **non-adherence.**
Glucocorticoid-exercise engagement interactions	Anxiety	• **Fear** of physical symptoms (e.g., dyspnoea).<br>• Avoidance of **exertion.**<br>• Heightened **sensitivity to bodily cues**.
	Depression	• **Low intrinsic motivation**.<br>• Persistent **apathy**.<br>• **Negative perception** of exercise.
	Neuropsychiatric symptoms	• **Unpredictable** mood or behaviour.<br>• Potential **safety risks** during unsupervised activity.
	Increased adiposity	• **Low self-efficacy**.<br>• Body image **dissatisfaction.**<br>• **Social withdrawal** from group exercise contexts.
Glucocorticoid-exercise adaptation interactions	Myopathy	• Decreased **muscle** mass and strength.<br>• Reduced fatigue **tolerance**.<br>• **Blunted responses** to PRT.
	Hyperglycemia/ Insulin resistance	• Impaired muscle **glucose uptake**.<br>• Reduced **anabolic signaling**.<br>• Increased **oxidative stress.**
	Weight gain/ Metabolic dysregulation	• Lower **aerobic efficiency.**<br>• **Difficulty progressing** intensity.<br>• **Altered energy balance** response.
	Bone turnover suppression	• **Reduced osteogenic response**.<br>• **Delayed adaptation** to mechanical loading.

5. Supplementary Table S2. Tailored exercise recommendations for managing glucocorticoid-induced adverse effects.

System	Condition/ Complication	Tailored exercise strategies
Musculo-skeletal	Osteoporosis	• Resistance training **2–3×/week** (focus on major muscle groups)<br>• Balance training 2×/week<br>• Guidance on safe movements (spine-sparing techniques)<br>• Emphasis on **spinal extensor strength**<br>• Avoid deep spinal flexion; use **spine-sparing techniques**<br>• **Individualised impact exercise** prescription based on pain, joint status, and fracture risk
	Myopathy	• Whole-body progressive resistance training<br>• Functional balance training to reduce fall risk<br>• Adherence to progressive overload principles
Cardiovas-cular	Hypertension	• Daily aerobic exercise at moderate intensity (40-60% of HRR), ≥30 min<br>• Resistance training **≥2×/week** at 40–60% 1RM<br>• Incorporate **extended cooldowns** and **proper breathing** (avoid Valsalva)<br>• **Monitor blood pressure** at rest and during exercise<br>• Observe for **alarm symptoms** (e.g., chest pain, dizziness)
Endocrine/ metabolic	Weight gain	• ≥150 **min/week** moderate- or ≥**60 min/week** vigorous-intensity activity<br>• Combined aerobic and resistance training<br>• Foster **autonomy** and **positive body image**<br>• Avoid stigmatizing language<br>• Set **realistic, health-centered goals** rather than focusing solely on weight loss
	Hyperglycaemia	• 150 min/week of moderate-to-vigorous aerobic exercise, spread over ≥3 days/week<br>• **≥2×/week** resistance training (major muscle groups)<br>• Emphasize **post-meal exercise timing**<br>• Include **frequent breaks** from sedentary behaviour throughout the day
Mental health	Anxiety	• Consider high-intensity training if appropriate<br>• Address autonomy, competence, and relatedness to enhance intrinsic motivation
	Depression	• Adapt training to **individual preferences**<br>• Prioritise **supervised, group-based, and recreational settings**
	Impaired cognitive function	• Machine-based resistance training (2×/week, 30–60’).<br>• **Power training** with embedded cognitive tasks (reaction-based)<br>• Use of **exergames** to enhance motivation and adherence

HRR: heart rate reserve

6. Supplementary Methods S1. Effects of glucocorticoids on exercise capacity, engagement, and physiological adaptations.

References for this section of the review were identified through searches of Pubmed, Web of Science, and Scopus. We performed one search for each glucocorticoid-induced effect, selecting the most relevant and representative articles of each search. The full search strategy for each effect was as follows:

- Osteoporosis: (glucocorticoid*[Title] OR glucocorticosteroid*[Title] OR corticosteroid*[Title] OR hydrocortisone[Title] OR cortisone[Title] OR prednisone[Title] OR prednisolone[Title] OR methylprednisolone[Title] OR betamethasone[Title] OR dexamethasone[Title] OR deflazacort[Title]) AND (osteoporosis[Title] OR bone[Title])
- Osteonecrosis: (glucocorticoid*[Title] OR glucocorticosteroid*[Title] OR corticosteroid*[Title] OR hydrocortisone[Title] OR cortisone[Title] OR prednisone[Title] OR prednisolone[Title] OR methylprednisolone[Title] OR betamethasone[Title] OR dexamethasone[Title] OR deflazacort[Title]) AND (osteonecrosis[Title] OR "avascular necrosis"[Title])
- Fractures: (glucocorticoid*[Title] OR glucocorticosteroid*[Title] OR corticosteroid*[Title] OR hydrocortisone[Title] OR cortisone[Title] OR prednisone[Title] OR prednisolone[Title] OR methylprednisolone[Title] OR betamethasone[Title] OR dexamethasone[Title] OR deflazacort[Title]) AND (fracture*[Title])
- Hypertension and cardiovascular risk: (glucocorticoid*[Title] OR glucocorticosteroid*[Title] OR corticosteroid*[Title] OR hydrocortisone[Title] OR cortisone[Title] OR prednisone[Title] OR prednisolone[Title] OR methylprednisolone[Title] OR betamethasone[Title] OR dexamethasone[Title] OR deflazacort[Title]) AND (hypertension[Title] OR "blood pressure"[Title] OR cardiovascular[Title])
- Cognitive/mood disorders: (glucocorticoid*[Title] OR glucocorticosteroid*[Title] OR corticosteroid*[Title] OR hydrocortisone[Title] OR cortisone[Title] OR prednisone[Title] OR prednisolone[Title] OR methylprednisolone[Title] OR betamethasone[Title] OR dexamethasone[Title] OR deflazacort[Title]) AND (anxiety[Title] OR depression[Title] OR mood[Title] OR cognitive[Title] OR psychiatric[Title] OR neuropsychiatric[Title])
- Weight gain: (glucocorticoid*[Title] OR glucocorticosteroid*[Title] OR corticosteroid*[Title] OR hydrocortisone[Title] OR cortisone[Title] OR prednisone[Title] OR prednisolone[Title] OR methylprednisolone[Title] OR betamethasone[Title] OR dexamethasone[Title] OR deflazacort[Title]) AND (dyslipidemia[Title] OR adipose[Title] OR "weight gain"[Title] OR fat[Title] OR "body composition"[Title])
- Myopathy: (glucocorticoid*[Title] OR glucocorticosteroid*[Title] OR corticosteroid*[Title] OR hydrocortisone[Title] OR cortisone[Title] OR prednisone[Title] OR prednisolone[Title] OR methylprednisolone[Title] OR betamethasone[Title] OR dexamethasone[Title] OR deflazacort[Title]) AND (myopathy[Title] OR muscle[Title] OR atrophy[Title])
- Falls: (glucocorticoid*[Title] OR glucocorticosteroid*[Title] OR corticosteroid*[Title] OR hydrocortisone[Title] OR cortisone[Title] OR prednisone[Title] OR prednisolone[Title] OR methylprednisolone[Title] OR betamethasone[Title] OR dexamethasone[Title] OR deflazacort[Title]) AND (fall*[Title/Abstract])
- Hyperglycaemia: (glucocorticoid*[Title] OR glucocorticosteroid*[Title] OR corticosteroid*[Title] OR hydrocortisone[Title] OR cortisone[Title] OR prednisone[Title] OR prednisolone[Title] OR methylprednisolone[Title] OR betamethasone[Title] OR dexamethasone[Title] OR deflazacort[Title]) AND (hyperglycemia[Title] OR hyperglycaemia[Title] OR insulin[Title] OR diabetes[Title] OR glucose[Title])

7. Supplementary Methods S2. Systematic review of RCTs assessing exercise interventions in adults taking oral glucocorticoids.

7.1. Search strategy

This systematic review with meta-analysis adhered to the guidelines outlined in the PRISMA (Preferred Reporting Items for Systematic Reviews and Meta-Analyses) statement [1]. It was registered in PROSPERO (CRD420251049238). The search for studies was conducted on May 10, 2025. Articles were gathered from Medline (PubMed), Web of Science, and Scopus. Two authors (PE-U and MLSA) independently performed systematic searches, and disagreements were resolved through discussion with a third author (MI). To standardize the quality and reporting of the studies, we established a methodological search filter to extract only the results published from 2000 onwards. Language filters were not used in this study. The full search strategy was as follows: (exercise[Title] OR training[Title] OR "physical activity"[Title]) AND (glucocorticoid* OR glucocorticosteroid* OR corticosteroid* OR cortisone OR prednisone OR prednisolone OR methylprednisolone OR dexamethasone OR deflazacort OR "rheumatoid arthritis"[Title/Abstract] OR "polymyalgia rheumatica"[Title/Abstract] OR vasculitis[Title/Abstract] OR "inflammatory myopath*"[Title/Abstract] OR polymyositis[Title/Abstract] OR dermatomyositis[Title/Abstract] OR "systemic lupus erythematosus"[Title/Abstract] OR "inflammatory bowel disease"[Title/Abstract] OR "ulcerative colitis"[Title/Abstract] OR "Crohn's disease"[Title/Abstract] OR "autoimmune hepatitis"[Title/Abstract] OR sarcoidosis[Title/Abstract]) AND (control*[Title/Abstract] OR random*[Title/Abstract]).

7.2. Selection criteria

The inclusion criteria were defined using the PICOS model (Population, Intervention, Comparison, Outcome, Study type) [2]. Articles were included if they met the following criteria: (a) included adults 18 years and over with autoimmune diseases, with at least half of them taking oral glucocorticoids; (b) evaluated a physical exercise intervention, including aerobic or resistance training; (c) included a group following usual care or participating in general care interventions; (d) assessed outcomes related to glucocorticoid-induced adverse effects; and (e) were randomized controlled trials (RCT). We did not include reviews, meeting abstracts, conference proceedings, or editorials. Additionally, we excluded studies that compared two structured exercise programs.

The search strategy gathered results from three databases. In addition, the reference lists of the most relevant articles were checked. Then, duplicates were eliminated. Initially, articles were screened by title and abstract, and studies that did not meet the inclusion criteria were excluded after a full-text review.

7.3. Data analysis

Two authors (PE-U and MLSA) independently extracted the summary data based on key observations. We recorded the following information for each study: study information (e.g., author name), population (e.g., age and number of participants), intervention (e.g., settings, supervision level, duration, and type of exercise), measurement methods, and outcomes (e.g., physical function, cognitive function, quality of life, risk of readmission or mortality, and cost-effectiveness).

Two authors (PE-U and MLSA) independently evaluated the risk of bias for each trial using the Risk of Bias 2 (RoB2) tool [3].

8. Supplemental Methods S3. Exercise recommendations.
The search for studies was conducted on May 10, 2025. Articles published from 2000 onwards were gathered from Medline (PubMed), Web of Science, and Scopus. We performed one search for each glucocorticoid-induced adverse effect, selecting the most relevant and representative articles of each search. The full search strategy for each effect was as follows:

- Osteoporosis, fractures and falls: (exercise[Title] OR "physical activity"[Title] OR training[Title]) AND (guidelines[Title] OR recommendations[Title] OR prescription[Title] OR consensus[Title] OR position stand[Title]) AND (osteoporosis[Title] OR fall*[Title] OR fracture*[Title] OR bone[Title])
- Myopathy: (exercise[Title] OR "physical activity"[Title] OR training[Title]) AND (guidelines[Title] OR recommendations[Title] OR prescription[Title] OR consensus[Title] OR position stand[Title] OR statement[Title]) AND (myopathy[Title] OR sarcopenia[Title] OR muscle[Title] OR strength[Title] OR fall*[Title])
- Hypertension and cardiovascular risk: (exercise[Title] OR "physical activity"[Title] OR training[Title]) AND (guidelines[Title] OR recommendations[Title] OR prescription[Title] OR consensus[Title] OR position stand[Title] OR statement[Title]) AND (hypertension[Title] OR cardiovascular[Title])
- Increased adipose tissue: (exercise[Title] OR "physical activity"[Title] OR training[Title]) AND (guidelines[Title] OR recommendations[Title] OR prescription[Title] OR consensus[Title] OR position stand[Title] OR statement[Title]) AND (fat[Title] OR "body composition"[Title] OR weight[Title])
- Hyperglycaemia: (exercise[Title] OR "physical activity"[Title] OR training[Title]) AND (guidelines[Title] OR recommendations[Title] OR prescription[Title] OR consensus[Title] OR position stand[Title] OR statement[Title]) AND (hyperglycemia[Title] OR hyperglycaemia[Title] OR insulin[Title] OR diabetes[Title])
- Mood disorders: (exercise[Title] OR "physical activity"[Title] OR training[Title]) AND (guidelines[Title] OR recommendations[Title] OR prescription[Title] OR consensus[Title] OR position stand[Title] OR statement[Title]) AND (mood[Title] OR depression[Title] OR anxiety[Title])
- Cognitive function: (exercise[Title] OR "physical activity"[Title] OR training[Title]) AND (guidelines[Title] OR recommendations[Title] OR prescription[Title] OR consensus[Title] OR position stand[Title] OR statement[Title]) AND (cognitive[Title] OR executive[Title])

9. References.

1. Page MJ, McKenzie JE, Bossuyt PM et al. The PRISMA 2020 statement: an updated guideline for reporting systematic reviews. BMJ. 2021;372:71; https://doi.org/10.1136/bmj.n71
2. Methley AM, Campbell S, Chew-Graham C, McNally R, Cheraghi-Sohi S. PICO, PICOS and SPIDER: a comparison study of specificity and sensitivity in three search tools for qualitative systematic reviews. BMC Health Serv Res. 2014;14:579; https://doi.org/10.1186/s12913-014-0579-0
3. Sterne J A C, SavoviÄ‡ J, Page M J, Elbers R G, Blencowe N S, Boutron I et al. RoB 2: a revised tool for assessing risk of bias in randomised trials BMJ. 2019; 366:l4898; https://doi.org/10.1136/bmj.l4898
